# Case Report: Long-term maternal and neonatal outcomes after nivolumab therapy in metastatic tonsillar sarcomatoid squamous cell carcinoma

**DOI:** 10.3389/fimmu.2026.1826390

**Published:** 2026-06-16

**Authors:** Elisabetta Guida, Benedetta Apollonio, Chiara Bungaro, Fabio Mele, Sabino Strippoli, Michele Guida

**Affiliations:** 1 Biostatistics and Bioinformatics Laboratory, IRCCS Istituto Tumori “Giovanni Paolo II”, Bari, Italy; 2 Rare Tumors and Melanoma Unit, IRCCS Istituto Tumori “Giovanni Paolo II”, Bari, Italy; 3 Pathology Department, IRCCS Istituto Tumori “Giovanni Paolo II”, Bari, Italy

**Keywords:** immune checkpoint inhibitor (ICI), immunotherapy, nivolumab, pregnancy, squamous cell carcinoma

## Abstract

**Background:**

Immunotherapy with checkpoint inhibitor (ICI) has revolutionized the treatment of cancer including squamous cell carcinoma. Nevertheless, there is an unmet need to gain a better understanding of the effect of these therapies on pregnancy and fertility. Treatment with anti-PD-1 therapy decreases fetal–maternal tolerance and increases the risk of pregnancy loss in animal studies. However, few data are currently available in humans.

**Case presentation:**

We report the case of conception and pregnancy, with successful maternal and fetal outcomes, in a young woman with metastatic squamous cell carcinoma originated from the palatin tonsil. She was 17-year-old at the diagnosis. After surgical removal of the primary tumor, she received adjuvant radiotherapy. Four years later she underwent surgery for a lung relapse and adjuvant chemotherapy. Three years later the patient experienced a pulmonary and mediastinal relapse. Immunotherapy with nivolumab was promptly started with a slow reduction of the disease until a near radiological complete response with a unique residual lung subpleural nodulation. Surgical romoval confirmed the residual disease of squamous cell carcinoma. Patient re-started immunotherapy with nivolumab but after 2 months, she was found to be 4 weeks pregnant. Treatment was stopped and, since the patient expressed her desire to carry her pregnancy to term, she was referred to gynecological and psychological support. She had an uneventful pregnancy, followed by spontaneous vaginal delivery. The baby was healthy and exhibited normal development. Furthermore, the patient breastfed until the sixth month without any adverse effects on the child growth. In November 2025, after 32 months of disease-free survival, the patient developed a new lung recurrence, presenting as a single 4-cm diameter lesion in the right lower lobe. In January 2026, she underwent surgical resection of the lesion and histology was consistent with the diagnosis of metastatic disease. Currently, in the absence of detectable disease, the patient continues active follow-up.

**Conclusions:**

Our case provides evidence that pregnancy and normal fetal development may occur after early exposure to nivolumab, Nevertheless, the impact of treatment interruption and pregnancy on disease recurrence remains an unresolved question.

## Introduction

Immunotherapy with checkpoint inhibitors (ICIs) has revolutionized the treatment of several types of cancer, including squamous cell carcinoma (1–3).

Despite excellent clinical results, there are still many uncertainties on the side effects of these drugs and their management (4). Moreover, the impact of immunotherapy on reproductive functions, including fertility, pregnancy, and lactation remains largely unknown (5). Although the incidence of cancer during pregnancy remains relatively low (about 1 in 1000 pregnancies (6)), most clinical trials include patients of reproductive age. In these patients, the use of contraceptive methods is recommended during ICI treatment and for a few months after the end of therapy. The Food and Drug Administration (FDA) categorized anti-PD-1 agents like nivolumab and pembrolizumab as pregnancy category D. This means that there is evidence of human fetal risk based on experimental or marketing experience, but the potential benefits of using the drug in pregnant women may justify its use in life-threatening conditions (7). In addition, the National Comprehensive Cancer Network (NCCN) guidelines strongly advise that patients of reproductive age should use effective birth control during and for at least 5 months after immunotherapy (8). Despite these recommendations, patient adherence to the use of prophylactic methods is not always considered, especially in clinical practice.

Animal studies have demonstrated that ICIs can reduce both female and male fertility through the induction of primary or secondary hypogonadism (9, 10).

The effects of ICIs on fetal development during pregnancy are less known. Immune checkpoints and their ligands play a crucial role in maternal-fetal immune tolerance and regulate the magnitude of immune responses during pregnancy (11). Studies in mice have demonstrated that the use of ICIs during pregnancy blocks the PD-1-PD-L1 axis resulting in a fivefold increased risk of fetal loss (12). Furthermore, as ICIs are IgG antibodies, they can cross the placenta and be transferred to the fetus, potentially leading to immune-related adverse events (13). Consequently, ICI-related side effects may be most pronounced during the third trimester (14). In addition, the use of ICIs during pregnancy might trigger irAEs in offspring, even several months after baby birth (15).

Notwithstanding, there is a lack of systematic studies characterizing frequency, duration, and presence of permanent alterations. A cohort study by Gougis et al. evaluating 91 pregnant individuals exposed to ICIs (including anti-PD-1/anti-PD-L1, combination therapy, or anti-CTLA-4 alone) showed no overall increase in adverse pregnancy, fetal, or neonatal outcomes compared to other anticancer drugs. However, combination therapy (anti-PD-1 plus anti-CTLA-4) was associated with a higher rate of preterm birth, a risk not seen with anti-PD-L1 or anti-CTLA-4 monotherapies. Nonetheless, due to the potential risk of rare immune-related neonatal adverse events, the authors concluded that ICI use during pregnancy should be avoided whenever possible (16). Furthermore, the mechanisms underlying the potential harmful impacts of these therapies on both the mother and fetus during gestation remain to be elucidated. Regulatory T (Treg) cells are well known to play essential roles in embryo implantation, placentation, the induction of maternal–fetal tolerance, and overall pregnancy progression. Physiologically, their primary function is to suppress immune cell-mediated inflammatory responses, preventing an overactivated maternal immune attack and thereby promoting fetal development (12, 17). Although considerable research has focused on the role of immune checkpoint molecules during pregnancy, their specific modulatory effects on gestational Treg cells remain largely unclear, and targeted clinical studies in pregnant women are currently lacking. Interestingly, the expression of key immune checkpoint molecules, including CTLA-4, PD-1, Tim-3, LAG-3, and TIGIT, is also characteristic of tumor-infiltrating Treg cells, where they are essential for maintaining suppressive capacity (17).

Even more limited data are available regarding the monitoring of neonatal growth and the maternal disease course following discontinuation of ICIs due to pregnancy. Furthermore, whether pregnancy itself may adversely affect disease recurrence after ICI cessation remains an unresolved question.

**Figure 1:**
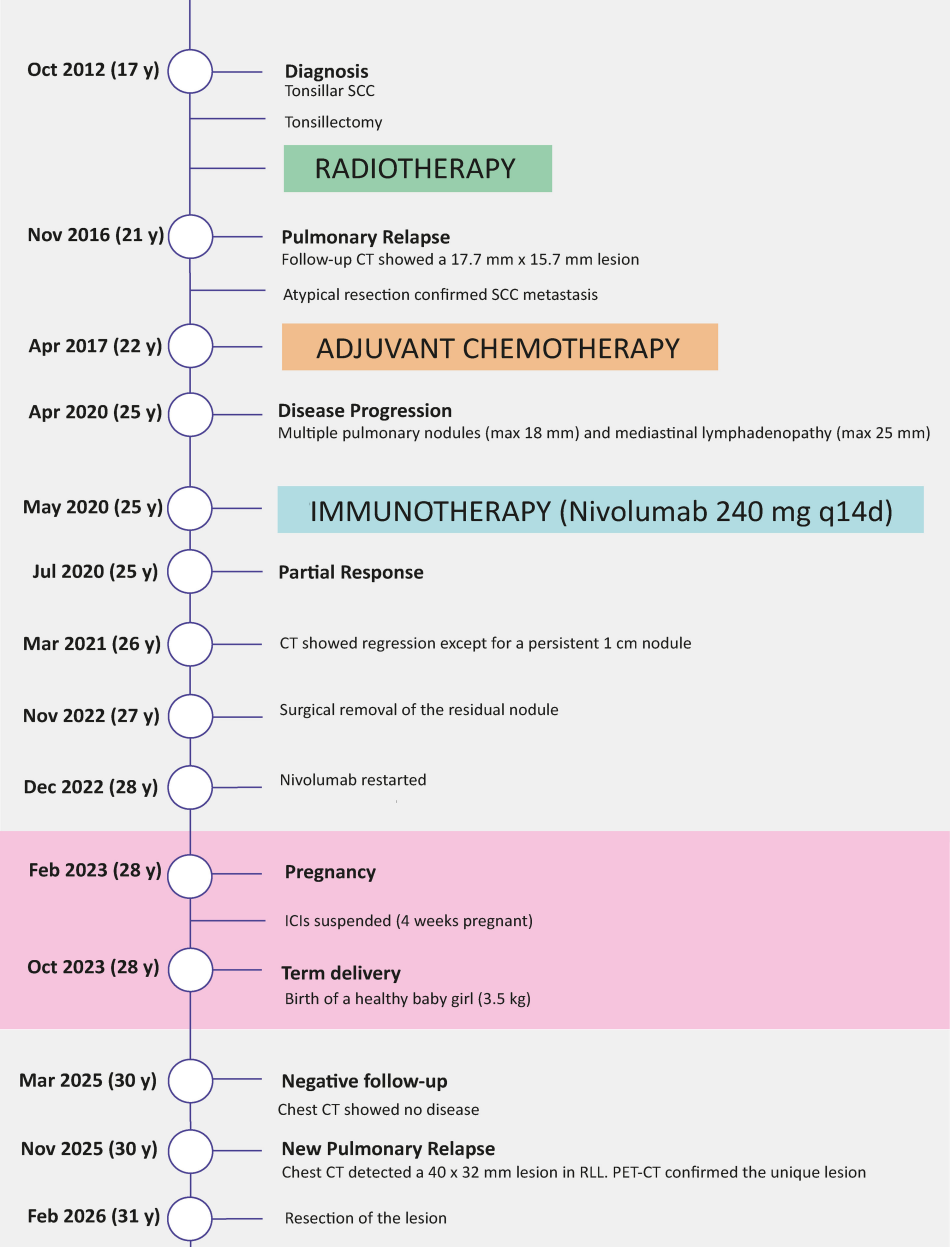
Timeline of the patient journey from the initial squamous cell carcinoma diagnosis.

Here, we report the case of a young woman who experienced a squamous cell carcinoma recurrence 32 months after discontinuing nivolumab due to pregnancy (**Figure 1**). We also discuss the subsequent management of her relapsed disease.

## Case presentation

### Clinical history

Woman, 31-year-old, non-smoker, without comorbidities nor neoplastic familiarity. In October 2012, at 17-years old, the patient presented with throat pain accompanied with a foreign body sensation. Following radiological and endoscopic examinations, she underwent surgical resection of left palatin tonsil. Histological analysis revealed a poorly differentiated papillomatous sarcomatoid squamous cell carcinoma (SCC) with high mitotic activity; immunohistochemistry revealed positivity for the p16 protein notoriously known to be related to HPV infection. The pathological stage was pT3 N0. Complementary radiotherapy (RT) was performed with 60 Gy.

**Figure 2:**
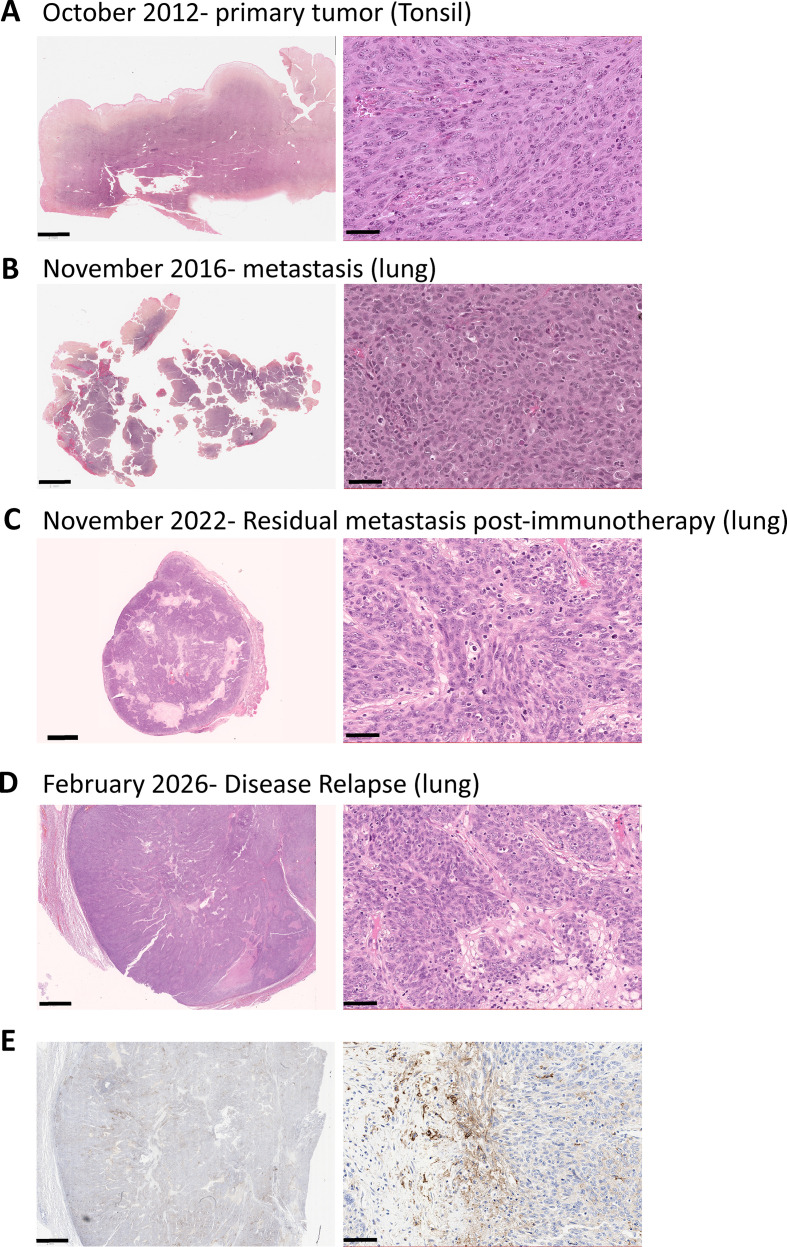
Hematoxilyn-eosin (H&E) staining of the primary tonsillar tumor [**(A)**, October 2012], the secondary disease localization [**(B)**, November 2016], the residual pulmonary lesion post-immunotherapy [**(C)**, November 2022], the disease relapse [**(D)**, February 2026]. **(E)** PD-L1 IHC staining on the disease relapse in February 2026. Scale bars: left panels 2mm, right panels 50μm.

In November 2016 (21-year-old) during the follow-up, CT scan showed a lung lesion of 17.7x15.7 mm at the right lower lobe. Due to the unicity of disease, the patient underwent pulmonary metastasectomy. Histopathological report and the revision of the first lesion confirmed the same poor differentiated SCC histology (**Figure 2A**). Subsequent adjuvant chemotherapy with 4 cycles of cisplatin and gemcitabine (Cisplatin 70mg/m^2^ and Gemcitabine 800mg/m^2^, administered on Days 1 and 8) was also administered.

**Figure 3:**
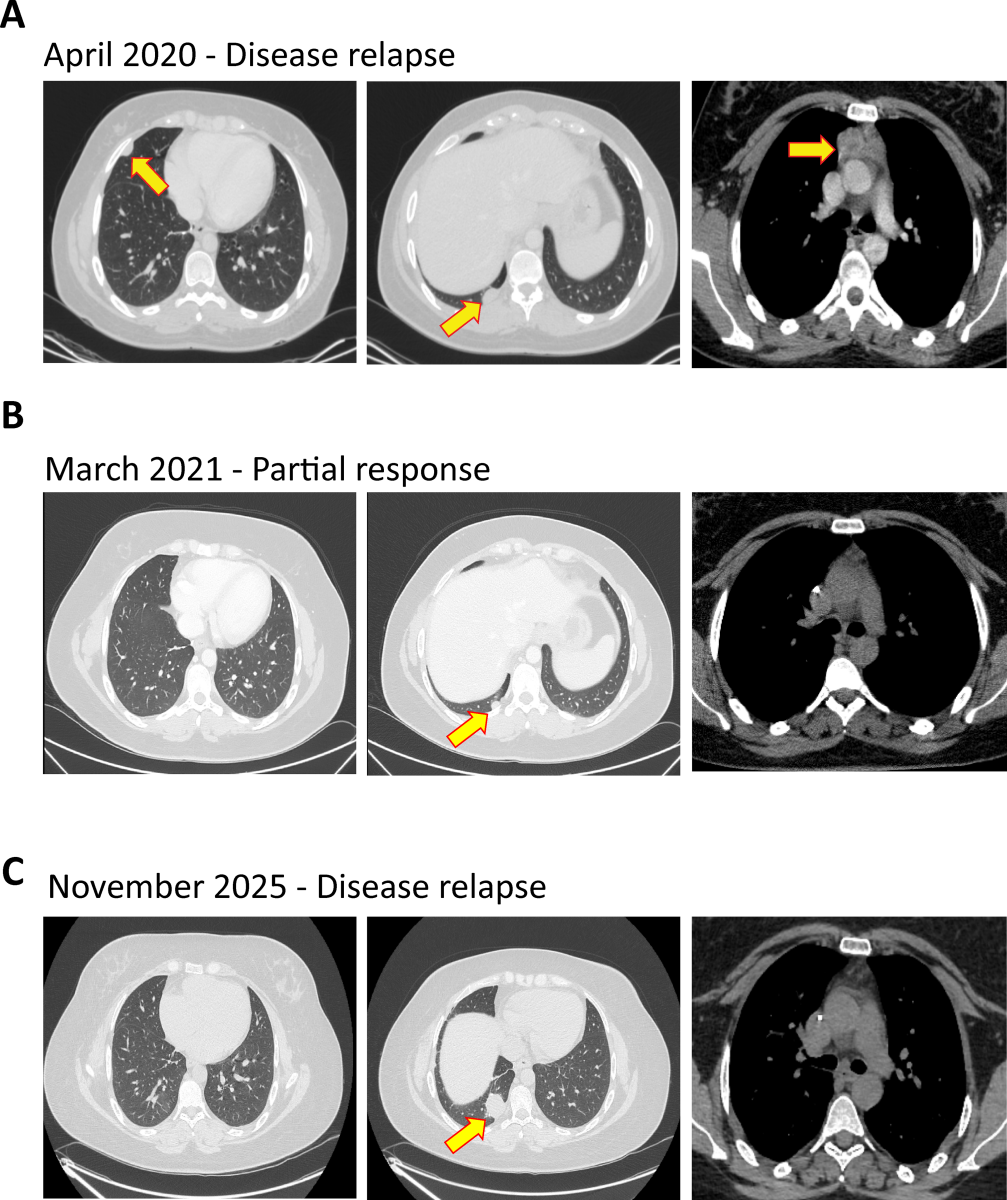
CT scans showing lung localization of the disease (arrows). **(A)** April 2020: subpleural nodules (left and central panels) and mediastinal adenophaty (right panel). **(B)** October 2021: residual subpleural nodule (central panel) after immunotherapy, while the previous frontal nodule (left panel), and mediastinal adenopathy (right panel) regressed. **(C)** November 2025: Pulmonary relapse 21 months after immunotherapy discontinuation. Yellow arrows point the different lesions.

In April 2020, during regular follow-up, a CT scan showed pulmonary nodules with a diameter max 18 mm at the right lower lobe and the presence of mediastinal adenopathic conglomerates of maximum 25 mm (**Figure 3A**, **Figure 2B**). After multidisciplinary tumor board discussion and in accordance with current clinical guidelines for recurrent/metastatic head and neck squamous cell carcinoma, treatment with the anti-PD-1 antibody nivolumab (240 mg intravenously every two weeks) was initiated (18, 19). Prior to treatment initiation, the patient was carefully counseled regarding potential immune-related adverse events and the need to adopt effective contraception during therapy. CT scan performed in July 2020 showed a partial response with reduction of nodules and of mediastinal adenopathy. Patient continued Nivolumab at the same timing and dose. A new CT scan performed in March 2021 showed an almost complete response of disease, except for a small residual parenchymal nodule of approximately 1 cm at the right lower lobe (**Figure 3B**). A subsequent PET-CT confirmed a low glucose uptake of the identified the lesion. Given the sustained systemic response to immunotherapy over 30 months, the presence of a solitary residual pulmonary lesion, and the feasibility of complete resection, the case was discussed at the multidisciplinary tumor board and a surgical approach was proposed and performed in November 2022. Histological examination confirmed the presence of a residual disease of squamous cell carcinoma partly necrotic and mixed with lymphocyte cells and phlogistic infiltration (**Figure 2C**); Ki67 was 35%.

After surgery, she resumed immunotherapy. In February 2023 (28 years old) she informed the medical team of her 5 weeks pregnancy and her decision to carry it to term. The multidisciplinary tumor board recommended discontinuation of ICI and referred the patient to gynecology specialists. She was informed of the need to discontinue treatment because of unpredictable risk to the fetus, as well as the uncertainty regarding disease progression following treatment discontinuation and in the context of pregnancy. Psychological support was also provided.

Fetal intrauterine growth was normal, and no immune-related adverse events or signs of immunization status were observed. The pregnancy reached full term, resulting in the birth of a healthy 3.5 kg baby girl in October 2023. The mother breastfed for six months, and the child continued to thrive without complications. Long-term follow-up documented normal psychomotor development; at the moment, at 2 years and 7 months of age, the child weighs 18 kg and is in excellent health. Additionally, the mother successfully recovered normal gonadal function and regular menstrual cycles.

The patient remained under close clinical and radiological surveillance, with chest CT scans performed every six months. In November 2025, 32 months after nivolumab discontinuation, imaging revealed a new pulmonary lesion measuring 40 mm in the right lower lobe, consistent with disease recurrence (**Figure 3C**). A subsequent PET-CT scan confirmed the presence of the unique site of metabolic activity with an elevated FDG uptake (SUV 10.6). Re-evaluated in the multidisciplinary team, a new surgical resection was proposed. After informed consensus, surgery was performed in February 2026. Histological examination confirmed the diagnosis with a highly undifferentiated and aggressive pattern (high number of mitoses, Ki67 of 35%, **Figure 2D**). PD-L1 immunhistochemistry (IHC) revealed a combined positive score (CPS) of 15% (**Figure 2E**).

Currently, in the absence of detectable disease, the patient continues active follow-up.

## Discussion

ICIs have transformed the treatment landscape in clinical oncology, including squamous cell carcinoma, but their use in pregnancy remains a challenging area of unmet need (5).

Given the rarity of cancer during pregnancy and the very limited experience with immunotherapy in this setting, management within a multidisciplinary team and close monitoring of both the mother and the fetus throughout pregnancy and after delivery are strongly recommended. While chemotherapy administration during the first trimester is known to be clearly associated with an increased risk of miscarriage, congenital malformations, or even fetal death (20), the risks associated with ICIs during pregnancy remain poorly understood. Thus, treatment with immunotherapy during pregnancy should be carefully considered. Moreover, this risk is likely to vary among patients, as it might be influenced by the degree of fetal allogeneity and mother-fetal immune interaction.

During pregnancy, substantial adaptations occur in the maternal immune system to maintain protection against pathogens, while avoiding detrimental reactions to the semi-allogeneic fetus. The pathways involved in the establishment of this feto-maternal tolerance can be hijacked by tumors. For this reason, treatment with anti-PD-1 and anti-PD-L1 monoclonal antibodies could be problematic during pregnancy. By blocking the interaction between PD-1 and PD-L1, ICIs enhance the functional activity of T cells towards tumour cells but simultaneously could abrogate the established feto-maternal immunotolerance. This mechanism could lead to adverse effects on pregnancy, as shown in several animal models and case reports. Similarly, by activating patient immune system with immunotherapy, a broad range of other immune-related adverse events can occur and negatively affect the fetus or impede a future desired pregnancy (12).

Regarding fertility, ICIs therapy could compromise reproductive capacity in both males and females through the induction of primary or secondary hypogonadism. Primary hypogonadism includes direct impairment of oogenesis and spermatogenesis, reduction of testosterone (erectile dysfunction, decreased testicular weight, decreased sperm production, inflammatory loss of spermatogenesis), reduction of estradiol, FSH, LH anti-Mullerian hormone (ovarian insufficiency, irregular menstrual cycle pattern and a lack of newly formed corpora lutea in the ovaries, premature menopause). Secondary hypogonadism mechanisms include damage in the hypothalamus or, more frequently, in the pituitary gland causing a reduced activation of the hypothalamic-pituitary-gonadal axis, often panhypopituitarism, with associated hypothyroidism and adrenal insufficiency. ICI activity within the ovary increases immune cell infiltration and inflammatory cytokines expression (e.g. tumor necrosis factor-α), diminishes the ovarian follicular reserve, impairing the ability of oocytes to mature and ovulate (21). The direct influence of ICI treatment on spermatogenesis has been demonstrated in a retrospective review of a small cohort of 13 patients with metastatic melanoma who developed infertility after ICIs therapy and subsequently died. Six of the 7 men (86%) had impaired spermatogenesis, including focal active spermatogenesis, hypospermatogenesis, and Sertoli-cell–only syndrome. In contrast, impaired spermatogenesis was observed only in two out of six untreated men (33%) (22). These results suggest that fertility preservation should be strongly considered for both women and men prior to initiation of immunotherapy.

The effects of ICIs in pregnancy are more known and consistent. In cynomolgus monkeys, nivolumab administration during pregnancy resulted in spontaneous abortion and increased neonatal death when animals received between 9- and 42-times higher dose than the one administered in humans (12). During pregnancy blocking the interaction of PD-1 and PD-L1 increased the risk of fetal loss in mice (12). Also, either CTLA-4- or Tim-3 blocking antibodies caused greater susceptibility to fetal loss in pregnant mice with altered cytokine profiles by decidual CD4^+^T cells (23). Moreover, ICIs are regarded as IgG antibodies capable of crossing the placenta, particularly during the third trimester, resulting in fetal exposure (13). Therefore, ICI-related side effects may be most noticeable during the third trimester (14).

In humans, ICI therapy has been reported to potentially induce miscarriage or alter the development and function of the fetal immune system (24, 25). The risk of adverse fetal outcomes seems likely mediated more by the maternal immune response to the fetus than by direct cytotoxic effects of the therapy. Consequently, this risk varies considerably between patients, as both feto-maternal immune tolerance and PD-L1 expression at the utero–placental interface influences the degree of fetal allogenicity. A recent systemic review of seven studies summarized all available data on ICIs exposure during human pregnancy (26). The mean gestational age at delivery resulted 30.4 weeks, whereas the mean weight of neonates at delivery was 1267 grams. Only one neonate was born term at 38 weeks of pregnancy; moreover, complications during pregnancy were observed in 71.4% of cases including intrauterine growth restriction (three cases), HELLP syndrome (hemolysis, elevated liver enzymes, low platelet count) (one case), placental insufficiency (one case) and low fetal heart rate (one case). The mean progression-free survival and overall survival were 16.0 and 25.2 months, respectively. The Authors concluded that administration of ICI during pregnancy is associated with increased incidence of pregnancy complications, prematurity and low birth weight (26). Anti-CTLA-4 treatment also resulted in maternal weight loss, increased rate of miscarriage, foetal death and preterm birth, with these adverse reactions observed mainly in the third trimester (10). Notably, the use of ICIs during pregnancy might trigger irAEs in offspring, even several months after birth. It has been reported a case of severe immune-related gastroenterocolitis in a 4-month-old infant who presented with intractable diarrhea and failure to thrive after *in utero* exposure to pembrolizumab (15). Finally, particular attention should be paid to patients with metastatic disease due to the risks of placental or fetal metastasis, although little is known about placental and fetal metastasis among pregnant women with cancer (27).

Clear evidence of the potential harmful effects of breastfeeding during ICIs treatment on infants is not available, however some Authors reported a cumulative increase of ICIs concentrations in the breastmilk birth. Although breastmilk concentrations were substantially lower than serum concentrations, the active drug could be detected up to 3 weeks after the final infusion. The amount of ipilimumab in breastmilk appears to be very low, but it may increase with subsequent doses during a treatment cycle. It is likely to be partially destroyed in the infant gastrointestinal tract and absorption by the infant is probably minimal (28).

Our patient, despite being treated with nivolumab until 4 weeks of pregnancy, reported no significant side effects. Fetal growth was also regular, as was growth after birth. Currently the child is 2-years and 7 months old, and she is in good health and her growth is regular.

Due to the clinical complexity of these cases, management should be guided by specialized multidisciplinary tumour boards, involving not only medical oncologists but also perinatal and pediatric specialists. In patients with metastatic disease, even after achieving a complete response, careful monitoring for recurrence is required. Indeed, the risk of recurrence for the mother after pregnancy remains elevated, particularly in patients with an aggressive histology or in those who have not achieved a durable complete response.

This case also highlights the importance of a multidisciplinary approach in the management of the oligometastasis. The decision to integrate pulmonary metastasectomy after a durable systemic response to immunotherapy was carefully discussed within a multidisciplinary tumor board and supported by available evidence and guideline recommendations suggesting a potential role for metastasis-directed therapy in selected patients with oligometastatic head and neck cancer (5, 29, 30). However, whether continuation or re-initiation of immunotherapy after surgical resection of residual disease could further consolidate the response and reduce the risk of relapse remains an open clinical question. Despite an excellent response to ICI, our patient experienced disease relapse 32 months after therapy discontinuation. The early discontinuation of therapy due to the pregnancy (only two months post-surgery) was likely insufficient to ensure durable disease control and prevent recurrence. In this regard, it is known that clinical benefits of ICI therapy may be long-lasting and could persist even after discontinuation, but the optimal treatment duration in metastatic disease has not be fully determined yet. In recurrent or metastatic head and neck squamous cell carcinoma, the optimal duration of anti-PD-1 therapy after achieving disease control remains unclear. In the pivotal clinical trials leading to the approval of nivolumab, treatment was typically continued until disease progression or unacceptable toxicity, and current guidelines do not provide specific recommendations regarding elective discontinuation in long-term responders. In a recent study addressing the timing of nivolumab discontinuation in long-term responders, progression-free survival appeared to reach a plateau at approximately three years, raising the hypothesis that prolonged therapy may be necessary to consolidate durable responses. This contrasts with data from other malignancies, for exsample in metastatic melanoma, in which elective discontinuation after around two years of therapy has been associated with sustained disease control and long-lasting responses. However, real life experiences reported that recurrences were observed in approximately two-thirds of cases among patients who did not achieve a complete response (25, 26).

## Conclusion

The limited data regarding patients treated with ICI during pregnancy highlight the need for cautious administration of these drugs during pregnancy. Although the risk for pregnancy and fetal development is relatively low, the risk of recurrence for the mother after pregnancy remains elevated, especially when a complete and lasting response has not been achieved. Also, if and how pregnancy could influence the recurrence and aggressiveness of the disease remains an unanswered question.

## Data Availability

The raw data supporting the conclusions of this article will be made available by the authors, without undue reservation.

## References

[1] Ghahremanloo A Soltani A Modaresi SMS Hashemy SI . Recent advances in the clinical development of immune checkpoint blockade therapy. Cell Oncol (Dordr). (2019) 42:609–26. doi: 10.1007/s13402-019-00456-w 31201647 PMC12994343

[2] Larkin J Chiarion-Sileni V Gonzalez R Grob JJ Rutkowski P Lao CD . Five-year survival with combined nivolumab and ipilimumab in advanced melanoma. N Engl J Med. (2019) 381:1535–46. doi: 10.1056/nejmoa1910836 31562797

[3] Aboaid H Khalid T Hussain A Myat YM Nanda RK Srinivasmurthy R . Advances and challenges in immunotherapy in head and neck cancer. Front Immunol. (2025) 16:1596583. doi: 10.3389/fimmu.2025.1596583 40547025 PMC12179090

[4] Wang DY Johnson DB Davis EJ . Toxicities associated with PD-1/PD-L1 blockade. Cancer J. (2018) 24:36–40. doi: 10.1097/ppo.0000000000000296 29360726 PMC5784852

[5] Helgadottir H Matikas A Fernebro J Frödin JE Ekman S Rodriguez-Wallberg KA . Fertility and reproductive concerns related to the new generation of cancer drugs and the clinical implication for young individuals undergoing treatments for solid tumors. Eur J Cancer. (2024) 202:114010. doi: 10.1016/j.ejca.2024.114010 38520926

[6] de Haan J Verheecke M Van Calsteren K Van Calster B Shmakov RG Mhallem Gziri M . Oncological management and obstetric and neonatal outcomes for women diagnosed with cancer during pregnancy: a 20-year international cohort study of 1170 patients. Lancet Oncol. (2018) 19:337–46. doi: 10.1016/s1470-2045(18)30059-7 29395867

[7] https://www.accessdata.fda.gov/drugsatfda_docs/label/2020/125377s110lbl.pdf (2026). Available online at: https://www.accessdata.fda.gov/drugsatfda_docs/label/2020/125377s110lbl.pdf (Accessed May 2020).

[8] National Comprehensive Cancer Network (NCCN) PM, PA, USA, 2025 . NCCN clinical practice guidelines in oncology: melanoma version 2. In: NCCN clinical practice guidelines in oncology: melanoma (2025).

[9] Duma N Lambertini M . It is time to talk about fertility and immunotherapy. Oncologist. (2020) 25:277–8. doi: 10.1634/theoncologist.2019-0837 32091651 PMC7160400

[10] Garutti M Lambertini M Puglisi F . Checkpoint inhibitors, fertility, pregnancy, and sexual life: a systematic review. ESMO Open. (2021) 6:100276. doi: 10.1016/j.esmoop.2021.100276 34597942 PMC8487000

[11] Chen Z Huang J Kwak-Kim J Wang W . Immune checkpoint inhibitors and reproductive failures. J Reprod Immunol. (2023) 156:103799. doi: 10.1016/j.jri.2023.103799 36724630

[12] Guleria I Khosroshahi A Ansari MJ Habicht A Azuma M Yagita H . A critical role for the programmed death ligand 1 in fetomaternal tolerance. J Exp Med. (2005) 202:231–7. doi: 10.1084/jem.20050019 16027236 PMC2213002

[13] Xu W Moor RJ Walpole ET Atkinson VG . Pregnancy with successful foetal and maternal outcome in a melanoma patient treated with nivolumab in the first trimester: case report and review of the literature. Melanoma Res. (2019) 29:333–7. doi: 10.1097/cmr.0000000000000586 30730328

[14] Poulet FM Wolf JJ Herzyk DJ DeGeorge JJ . An evaluation of the impact of PD-1 pathway blockade on reproductive safety of therapeutic PD-1 inhibitors. Birth Defects Res B Dev Reprod Toxicol. (2016) 107:108–19. doi: 10.1002/bdrb.21176 27062127

[15] Baarslag MA Heimovaara JH Borgers JSW van Aerde KJ Koenen HJPM Smeets RL . Severe immune-related enteritis after in utero exposure to pembrolizumab. N Engl J Med. (2023) 389:1790–6. doi: 10.1056/nejmoa2308135 37937778

[16] Gougis P Hamy AS Jochum F Bihan K Carbonnel M Salem JE . Immune checkpoint inhibitor use during pregnancy and outcomes in pregnant individuals and newborns. JAMA Netw Open. (2024) 7:e245625. doi: 10.1001/jamanetworkopen.2024.5625 38630478 PMC11024778

[17] Zhang YH Sun HX . Immune checkpoint molecules in pregnancy: Focus on regulatory T cells. Eur J Immunol. (2020) 50:160–9. doi: 10.1002/eji.201948382 31953958

[18] Machiels JP René Leemans C Golusinski W Grau C Licitra L Gregoire V . Squamous cell carcinoma of the oral cavity, larynx, oropharynx and hypopharynx: EHNS-ESMO-ESTRO clinical practice guidelines for diagnosis, treatment and follow-up. Ann Oncol. (2020) 31:1462–75. doi: 10.1016/j.annonc.2020.07.011 33239190

[19] Ferris RL Blumenschein G Fayette J Guigay J Colevas AD Licitra L . Nivolumab for recurrent squamous-cell carcinoma of the head and neck. N Engl J Med. (2016) 375:1856–67. doi: 10.1056/nejmoa1602252 27718784 PMC5564292

[20] Cardonick E Iacobucci A . Use of chemotherapy during human pregnancy. Lancet Oncol. (2004) 5:283–91. doi: 10.1016/s1470-2045(04)01466-4 15120665

[21] Cacciottola L Camboni A Dolmans MM . Immune system regulation of physiological and pathological aspects of the ovarian follicle pool throughout the female reproductive lifespan. Hum Reprod. (2025) 40:12–22. doi: 10.1093/humrep/deae254 39607771

[22] Scovell JM Benz K Samarska I Kohn TP Hooper JE Matoso A . Association of impaired spermatogenesis with the use of immune checkpoint inhibitors in patients with metastatic melanoma. JAMA Oncol. (2020) 6:1297–9. doi: 10.1001/jamaoncol.2020.1641 32556068 PMC7303895

[23] Wang S Chen C Li M Qian J Sun F Li Y . Blockade of CTLA-4 and Tim-3 pathways induces fetal loss with altered cytokine profiles by decidual CD4. Cell Death Dis. (2019) 10:15. doi: 10.1038/s41419-018-1251-0 30622243 PMC6325160

[24] Borgers JSW Heimovaara JH Cardonick E Dierickx D Lambertini M Haanen JBAG . Immunotherapy for cancer treatment during pregnancy. Lancet Oncol. (2021) 22:e550–61. doi: 10.1016/s1470-2045(21)00525-8 34856152

[25] Lee CL Martinez E Malon Gimenez D Muniz TP Butler MO Saibil SD . Female oncofertility and immune checkpoint blockade in melanoma: Where are we today? Cancers (Basel). (2025) 17. doi: 10.22541/au.172320180.09605335/v1 39858020 PMC11763405

[26] Andrikopoulou A Korakiti AM Apostolidou K Dimopoulos MA Zagouri F . Immune checkpoint inhibitor administration during pregnancy: a case series. ESMO Open. (2021) 6:100262. doi: 10.1016/j.esmoop.2021.100262 34487972 PMC8426195

[27] Khazzaka A Rassy E Sleiman Z Boussios S Pavlidis N . Systematic review of fetal and placental metastases among pregnant patients with cancer. Cancer Treat Rev. (2022) 104:102356. doi: 10.1016/j.ctrv.2022.102356 35182890

[28] Anderson PO . Monoclonal antibodies during breastfeeding. Breastfeed Med. (2021) 16:591–3. doi: 10.1089/bfm.2021.0110 33956488

[29] Shiono S . The role of pulmonary metastasectomy for pulmonary metastasis from head and neck cancer. J Thorac Dis. (2021) 13:2643–8. doi: 10.21037/jtd.2020.04.14 34012613 PMC8107534

[30] Matsuo M Masuda M Yamauchi M Hashimoto K Kogo R Sato M . Progression-free survival and treatment-free interval in head and neck cancer with long-term response to nivolumab: Timing of active discontinuation. Cancers (Basel). (2024) 16. doi: 10.20944/preprints202406.1840.v1 PMC11274866 39061167

